# Supplementary material for: Identification of photoperiod-induced specific miRNAs in the adrenal glands of Sunite sheep (Ovis aries)
Source: Front Vet Sci. 2022 Jul 22;9:888207. doi: 10.3389/fvets.2022.888207 (PMC9354845; doi:10.3389/fvets.2022.888207)
Supplement: Supplementary file 1 [file Data_Sheet_1.ZIP › Supplementary file/miRDeep2 file.zip!/miRDeep2 file/Novel_Structure/SPLP42A2/Novel_154.pdf]

Provisional ID	:	NC_019459.2_5122
Score total	:	2.2
Score for star read(s)	:	-1.3
Score for read counts	:	0
Score for mfe	:	1.9
Score for randfold	:	1.6
Score for cons. seed	:	
Total read count	:	151
Mature read count	:	151
Loop read count	:	0
Star read count	:	0

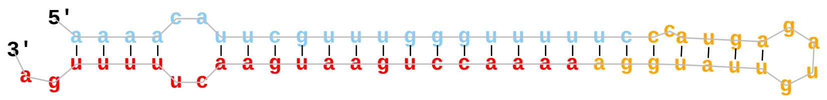

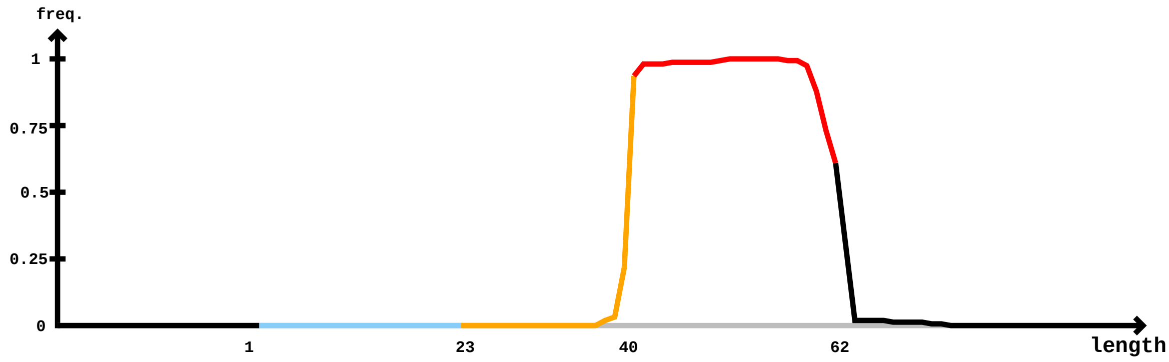

Star Mature

```
5'- uuuaaauauaggguuggacaaaacauucguuuggguuuuucccaugagauguuauggaaaaaccugaaugaacuuuuugaccaacccaauauuuuggguuuagagguuuaa -3'    exp
    ...((((((.(((((((.(((((..((((((((((((((((.((((....))))))))))))))))))))..))))).))))))).))))))...................    reads    mm    sample
    .......................................................ggaaaaaccugaaugaacuuu...................................    1    0    seq
    .......................................................Ugaaaaaccugaaugaacuuuu..................................    1    1    seq
    .......................................................ggaaaaaccugaaugaacuuuu..................................    1    0    seq
    ........................................................gaaaaaccugaaugaacuuC...................................    1    1    seq
    ........................................................gaaaaaccAgaaugaacuuuu..................................    1    1    seq
    .........................................................aaaaaccugaaugaaUu.....................................    1    1    seq
    .........................................................aaaaaccugaaGgaacuuuu..................................    1    1    seq
    .........................................................aaaaaccAgaaugaacuuuu..................................    10    1    seq
    .........................................................aaaaaccugaaugaacuuuuC.................................    1    1    seq
    .........................................................Caaaaccugaaugaacuuuuu.................................    1    1    seq
    .........................................................aaaaaccugaaGgaacuuuuug................................    12    1    seq
    .........................................................aaaaaccugaaugaacuuuCug................................    1    1    seq
    .........................................................aaaaaccugaaugaacuuuuuga...............................    1    0    seq
    .........................................................aaaaaccugaaugaacuuuuugCc..............................    1    1    seq
    ..........................................................aaaaccAgaaugaacuuu...................................    1    1    seq
    ..........................................................aaaaccAgaaugaacuuuu..................................    1    1    seq
    ..........................................................aaaaccugaaugaacuuuuu.................................    13    0    seq
    ..........................................................aaaaccAgaaugaacuuuuu.................................    7    1    seq
    ..........................................................Uaaaccugaaugaacuuuuu.................................    1    1    seq
    ..........................................................aaaaccugaaugaacuuuuuA................................    1    1    seq
    ..........................................................aaaGccugaaugaacuuuuug................................    1    1    seq
    ..........................................................Gaaaccugaaugaacuuuuug................................    2    1    seq
    ..........................................................aaaaccugaaugaacuCuuuga...............................    1    1    seq
    ..........................................................aaaaccugaaugaacuuuuuga...............................    11    0    seq
    ..........................................................aaaaccugaaugaacuuuuugU...............................    7    1    seq
    ..........................................................aaaaUcugaaugaacuuuuuga...............................    66    1    seq
    ...........................................................aaaccugaaugaacuuuuug................................    1    0    seq
    ...........................................................aaaccAgaaugaacuuuuug................................    1    1    seq
    ...........................................................aaaccugaaugaacuuuuuga...............................    1    0    seq
    ...........................................................aaaccugaaugaacuuuuugU...............................    3    1    seq
    ...........................................................aaaccugaauAaacuuuuugac..............................    1    1    seq
    ..............................................................ccugaaugaacuuuuugaccaaU..........................    1    1    seq
    ...................................................................augaacuuuuugaccaacccaaAa....................    1    1    seq
    ....................................................................ugaacuuuuugCccaacccaa......................    1    1    seq
```

Star Mature

uuuaaauauaggguuggacaaaacauucguuuggguuuuucccaugagauguuauggaaaaaccugaaugaacuuuuugaccaacccaauauuuugguuuagagguuuaa
